# Binocular Neurons in Parastriate Cortex: Interocular ‘Matching’ of Receptive Field Properties, Eye Dominance and Strength of Silent Suppression

**DOI:** 10.1371/journal.pone.0099600

**Published:** 2014-06-13

**Authors:** Phillip A. Romo, Natalie Zeater, Chun Wang, Bogdan Dreher

**Affiliations:** Discipline of Anatomy and Histology, School of Medical Sciences & Bosch Institute, University of Sydney, New South Wales, Australia; University of Sussex, United Kingdom

## Abstract

Spike-responses of single binocular neurons were recorded from a distinct part of primary visual cortex, the parastriate cortex (cytoarchitectonic area 18) of anaesthetized and immobilized domestic cats. Functional identification of neurons was based on the ratios of phase-variant (F1) component to the mean firing rate (F0) of their spike-responses to optimized (orientation, direction, spatial and temporal frequencies and size) sine-wave-luminance-modulated drifting grating patches presented separately via each eye. In over 95% of neurons, the interocular differences in the phase-sensitivities (differences in F1/F0 spike-response ratios) were small (≤0.3) and in over 80% of neurons, the interocular differences in preferred orientations were ≤10°. The interocular correlations of the direction selectivity indices and optimal spatial frequencies, like those of the phase sensitivies and optimal orientations, were also strong (coefficients of correlation r ≥0.7005). By contrast, the interocular correlations of the optimal temporal frequencies, the diameters of summation areas of the excitatory responses and suppression indices were weak (coefficients of correlation r ≤0.4585). In cells with high eye dominance indices (HEDI cells), the mean magnitudes of suppressions evoked by stimulation of silent, extra-classical receptive fields via the non-dominant eyes, were significantly greater than those when the stimuli were presented via the dominant eyes. We argue that the well documented ‘eye-origin specific’ segregation of the lateral geniculate inputs underpinning distinct eye dominance columns in primary visual cortices of mammals with frontally positioned eyes (distinct eye dominance columns), combined with significant interocular differences in the strength of silent suppressive fields, putatively contribute to binocular stereoscopic vision.

## Introduction

In mammals with frontally positioned eyes such as domestic cats or macaque monkeys, the great majority (∼90%) of neurons in the dorsal lateral geniculate nucleus (LGNd) are reported to be monocular, that is, they generate action potentials (spikes) only when appropriate visual stimuli are presented via a particular eye [1]–[6]. In the striate cortices (cytoarchitectonic areas 17, areas V1) of those species, within topographic representation of a given part of the contralateral visual hemifield, there is very little overlap between the subregions of the geniculo-recipient layers which receive inputs from the LGNd neurons relaying signals from either the contralateral or the ipsilateral eyes [7]–[11]. This ‘eye-origin-specific’ spatial segregation of the geniculo-cortical terminals constitutes a putative basis for the so-called ‘eye dominance columns’ [7]. However, despite the ‘eye-origin-specific’ segregation of geniculo-cortical terminals which provide principal excitatory drive to the primary visual cortex, most neurons in the primary visual cortices are binocular, that is, they generate spikes when appropriate visual stimuli are presented in the topographically approximately corresponding parts of each retina.

Nearly two centuries ago, Wheatstone (1838) postulated that in humans, stereoscopic single binocular vision is derived from tiny differences in left and right images resulting from binocular parallax [12]. More recently, it has been argued that the mammalian stereoscopic single vision is largely based on the interocular differences in the position (disparities) of the discharge fields - position disparity model [13]–[16] and/or interocular differences in the spatial arrangement of receptive field subregions - phase-shift model [17]–[21] of binocular neurons in the primary visual cortices. Furthermore, it has been argued that both stereoscopic single vision and binocular depth discrimination are derivatives of binocular interactions (facilitatory and/or suppressive) at the single neuron level in the primary visual cortices [13], [14], [22]–[26].

Parastriate cortex (cytoarchitectonic area 18) of carnivores such as domestic cat, constitutes a distinct part of primary visual cortex, which like the striate cortex receives its principal direct dorsal thalamic input from the LGNd [27]–[29]. As in the case of area 17, the eye-specfic LGNd inputs to area 18 are spatially segregated and there are clear eye dominance columns in this area [9], [10], [30]–[34]. Despite this, the great majority of area 18 neurons, like the great majority of area 17 neurons (see above), are binocular [27], [28], [35]. Furthermore, a large proportion of cat's area 18 neurons, like a large proportion area 17 neurons, are sensitive to retinal disparities [36]–[39]. However, the mechanism of binocular interactions in most retinal disparity sensitive area 18 neurons might be quite different from that operating in most retinal disparity sensitive area 17 neurons. For example, so-called tuned-excitatory cells characterized by narrowly tuned binocular facilitation dominate area 17, while so-called near and far cells which are characterized by reduction or a complete suppression of spike-responses over a wide range of retinal disparities are common among area 18 neurons [38], [39]. It has been suggested that substantial degree of the interocular similarity of receptive field properties of binocular area 17 neurons [16], [40] underpin psychophysical fusion of the images formed in the topographically corresponding parts of the two retinae. However, very little is known about the extent of similarity (or otherwise) of receptive field properties of binocular area 18 neurons [41].

Thus, in the present study, using patches of sine-wave, luminance-contrast-modulated drifting gratings presented separately via each eye we assessed quantitatively the degree of interocular matching of a number of receptive field properties of binocular cells recorded from cat's area 18. We discuss the implications as well as putative mechanisms underlying good or poor interocular matching of particular receptive field properties in primary visual cortices in general. Furthermore, we propose that observed by us significant differences in the strength of silent suppression in receptive fields of the dominant and the non-dominant eyes of area 18, combined with segregation of geniculate inputs into distinct eye dominance columns might be of substantial use in stereoscopic vision.

## Materials and Methods

### 2.1 Animals, anaesthesia and surgical procedures

Experiments were carried out in accordance with the guidelines of the National Health and Medical Research Council's Code of Practice for the Care and Use of Animals for Scientific Purposes in Research in Australia and approved by the Animal Care Ethics Committee of the University of Sydney. Five females and one male adult domestic cats (*Felis catus*) weighing 2.5–5.0 kg, were supplied by Laboratory Animal Services of the University of Sydney. The animals were initially anesthetized with a gaseous mixture of 2–4% isoflurane in 65% N_2_O – 35% O_2_, and underwent a tracheal intubation and insertion of cannula into the cephalic vein. Throughout subsequent surgical procedures, isoflurane level in gaseous mixture was maintained at 0.75–1.5%. The animals were initially paralyzed with 2 ml bolus of gallamine triethiodide (40 mg/ml) followed by a continuous intravenous infusion of gallamine (10 mg/kg/h) throughout the rest of experiment. Potential residual eye movements were prevented by bilateral cervical sympathectomy [42]. Throughout the experiment the animals were artificially respired, with the end tidal CO_2_ level maintained at 3.7–4.0% range by adjusting the rate (range 18–24 strokes/min) and/or the stroke volume of the pulmonary pump. A “deep sleep” state, characterized by the presence of delta waves (∼0.5–4 Hz) in the electroencephalogram (EEG) was maintained throughout experiment. Body temperature was maintained at 37°C, monitored by a subscapular probe with an Animal Temperature Controller 1000 (World precision Instruments, FL, USA). A broad-spectrum antibiotic, 15 mg/kg amoxicillin trihydrate (150 mg/mL) and 0.05 mg/kg atropine sulfate (0.6 mg/mL) to reduce mucosal excretion, were administered intramuscularly daily. Topical daily application of a 1% atropine and 0.1% phenylephrine aqueous solution dilated the pupils, blocked accommodation and retracted the nictitating membranes. The animal's eyes were covered with zero-power, air permeable contact lenses. Artificial pupils (3 mm in diameter) and appropriate corrective lenses to focus the eyes on a tangent screen 57 cm away were positioned in front of the animal. The position of the optic discs were regularly (at least twice every 24 hours) back-projected using a fibre-optic light source [43] and the presumed position of the *areae centrales* (AC) were plotted directly and/or by reference to the position of the centers of optic discs [44], [45].

### 2.2 Recording single neuron activity from area 18

A craniotomy was performed and dura mater reflected over part of the left occipital lobe (Horsley-Clarke coordinates: 0–8 mm posterior and 2–9 mm lateral). A plastic chamber fitted to the craniotomy was glued to the skull, a stainless steel microelectrode (11 MΩ; FHC, ME, USA) was placed in the craniotomy, and the chamber was then filled with 4% agar and sealed with warm liquid wax. To assist with reconstruction of the electrode tracts, the microelectrode was coated with fluorescent dye - 1.1′-dioctatadecyl-3,3,3′3′-tetramethylindocarbocyanine perchlorate (DiI) [46] and then slowly advanced into area 18 using an electronic 8200 Inchworm motor controller (EXFO Photonic Solutions, ON, Canada). Action potentials (spikes) of single neurons were recorded extracellularly, amplified, band-pass filtered and monitored both visually (on an oscilloscope) and acoustically (via an audio monitor). The spikes identified by their unique shape and amplitude triggered standard pulses, which were fed into the computer for data collection. When recording the activity of a given cell the spikes were monitored continuously and testing was discontinued if triggering standard pulses was contaminated by the activity of other cells.

### 2.3 Assessment of sRF properties

A region in the visual space from which one can trigger spike-responses (action potentials) to appropriate visual stimuli at a rate exceeding that of background (‘spontaneous’) activity is defined here as the excitatory or minimum discharge receptive field. Summation receptive fields (sRF) are defined here as the regions stimulation of which with optimized (optimal orientation, direction, spatial and temporal frequencies) high-contrast (≥80%) grating patches result in the maximal spike discharge rate [29]. In most cells, the stimulation of region surrounding the sRF (without encroaching on sRF) did not produce action potentials (spikes) but was found to modulate the magnitude of spike-response of the cell when stimulated in conjunction with its sRF. This region is defined as the silent extra-classical receptive field (ECRF). The sRF diameters of neurons in cat's area 18 are usually ∼ three-fold greater than those of their minimum discharge fields and thus include not only the discharge fields but also part of the silent receptive field [29]. For each binocular neuron, the properties of both the sRF and the ECRF were assessed monocularly by presenting the stimuli through one eye while the other eye was covered with an occluder not transparent to light.

Achromatic sine-wave contrast modulated drifting gratings of variable size, orientation, and spatial and temporal frequency were generated by the visual stimulation system EXPO (P. Lennie, University of Rochester, NY, USA) and presented on a calibrated CRT monitor (Barco, West-Vlaanderen, Belgium) placed 57 cm in front of the animal. The mean luminance of the screen was held at ∼50 cd/m^2^, with the grating presented centred in a circular patch. Optimal orientation, spatial and temporal frequencies were determined at 100% Michelson contrast defined as 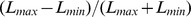 where 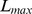 and 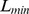 are, respectively, maximum and minimum luminance in the grating patch. Each stimulus was presented for 3 seconds, followed by a 1 second interstimulus interval of blank screen of mean luminance and no grating. A 3 sec recording at mean luminance was used to assess the level of background (‘spontaneous’) spike activity. Optimal orientation, optimal spatial frequency, optimal temporal frequency, contrast and optimal stimulus size as well as the tuning characteristics of these properties were determined in the assessment of the sRF stimulus that would produce the maximal response. Each series of visual stimuli was repeated 10 times and presented in a pseudo-randomized fashion.

### 2.4 Localization of recording sites

We are confident that all cells included in the present sample were located in area 18. Our confidence is based on the location of the discharge field within 5–10° from representation of the vertical meridian and typical area 18 receptive field properties [29], [47]. Furthermore, on occasions when we have made more medial penetrations closer to presumed area 17, the receptive fields of neurons encountered in these penetrations moved close to the representation of the vertical meridian the discharge fields became smaller and preferred higher spatial frequencies [28], [29], [48]. Finally, the areal location (area 18 vs. area 17) of recorded neurons was asessed histologically. To this purpose small electrolytic lesions (20 µA for 10 sec, electrode positive) were made at the end of the penetration and at two additional positions during retraction of the electrode. Each experiment was terminated via intravenous infusion of sodium pentobarbitone (120 mg/kg, Lethabarb). The animals were perfused transcardially with warm 40°C saline followed by 4% paraformaldehyde in phosphate buffer (0.1 M at pH 7.4). The appropriate region of the cortex has been frozen-sectioned and 50 µm coronal sections were stained with cresyl violet.

### 2.5 Data analysis and statistics

Peristimulus time histograms (PSTH) of spike-responses were constructed for each stimulus presentation and subjected to discrete Fourier analysis. Cells in which the phase-variant component of the response (F1) was greater than the mean firing rate F0, that is, F1/F0 spike-response ratio was >1, were classified as simple cells, those with an F1/F0 spike-response ratio <1 were classified as complex cells.

***Direction selectivity indices (DSI)*** of a given cell were calculated according to the following formula:
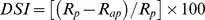



Where 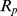
and 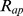 are the peak discharge rates for optimized sRF- confined stimulus moving in the preferred and anti-preferred (or null) directions respectively.

#### Spatial frequency tuning

Spatial frequency tuning profiles for sRF-confined stimuli were modelled using Gaussians model:
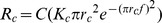



Where 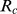 is the response amplitude, f is the spatial frequency of stimulus and 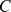 is the Michelson contrast of the stimulus. The free parameters 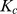
and 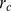
are centre peak sensitivity and centre Gaussian radius respectively.

#### Orientation tuning width

Orientation tuning was fit to a Gaussian according to the following equation: 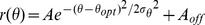



Where are 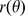 is the response amplitude at orientation 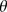. 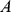 and 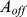 are the peak amplitude and amplitude offset respectively. 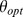 indicates the optimal orientation and 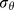 is the standard deviation of the Gaussian. The width of receptive field properties tuning, expressed as half-width at half-height (HWHH), is defined as the half the width of the Gaussian at half the peak height above the amplitude offset [49].

#### Assessment of eye dominance

The extent of response dominance by stimuli presented via one eye is expressed as the eye dominance index:
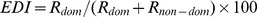



Where 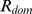 and 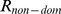, refer to the magnitude of response (in spikes/s) to the high (≥80%) - saturation level contrast, optimized stimuli presented via the dominant and non-dominant eye respectively [40].

#### Suppression index (SI)

The strength of suppression was assessed quantitatively and expressed as a suppression index, SI defined as:
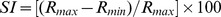



As the diameter of the optimized drifting grating patch was gradually increased from the initial size of 1°, the magnitude of response also gradually increased until it reached a maximum (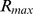). Consistent with our recent study of area 18 [29], in a proportion of cells further increases of the grating size resulted in substantial reduction of magnitude of response or even complete abolishment (suppression) of the response. In some cells however, further increases of the grating size resulted in no change in the magnitude of response (response saturation). The magnitude of response when the suppression was greatest was defined here as R_min_.

Statistical significance of the differences between the sets of data were assessed using nonparametric tests: Mann-Whitney U-test (referred to as a Mann-Whitney test and used to assess significance of differences of two independents sets of data), Wilcoxon matched-pairs signed-ranks test (referred to as Wilcoxon test and used to assess significance of differences of paired-data) and Spearman rank correlation coefficient test [50]. Values in the text indicate means ± the standard errors of the means (SEM). The statistical significance between the two sets of data unless otherwise indicated was accepted if the associated probability (P) value was 0.05 or less at the two-tailed criterion.

## Results

The present paper is based on quantitative analysis of spike-responses of 37 single binocular area 18 neurons, evoked by visual stimuli presented separately via each eye. The centers of their discharge fields were located within 20° from *areae centrales* (most - 24/37; 65% within 10° from *areae centrales*). The great majority (20/24; 85%) of neurons whose laminar location we were able to determine, were recorded either from the principal geniculo-recipient layer 4 or the geniculo-recipient, lower part of layer 3 - layer 3b [51], [52].

### 3.1 Identification of neurons as simple or complex

**Figure 1:**
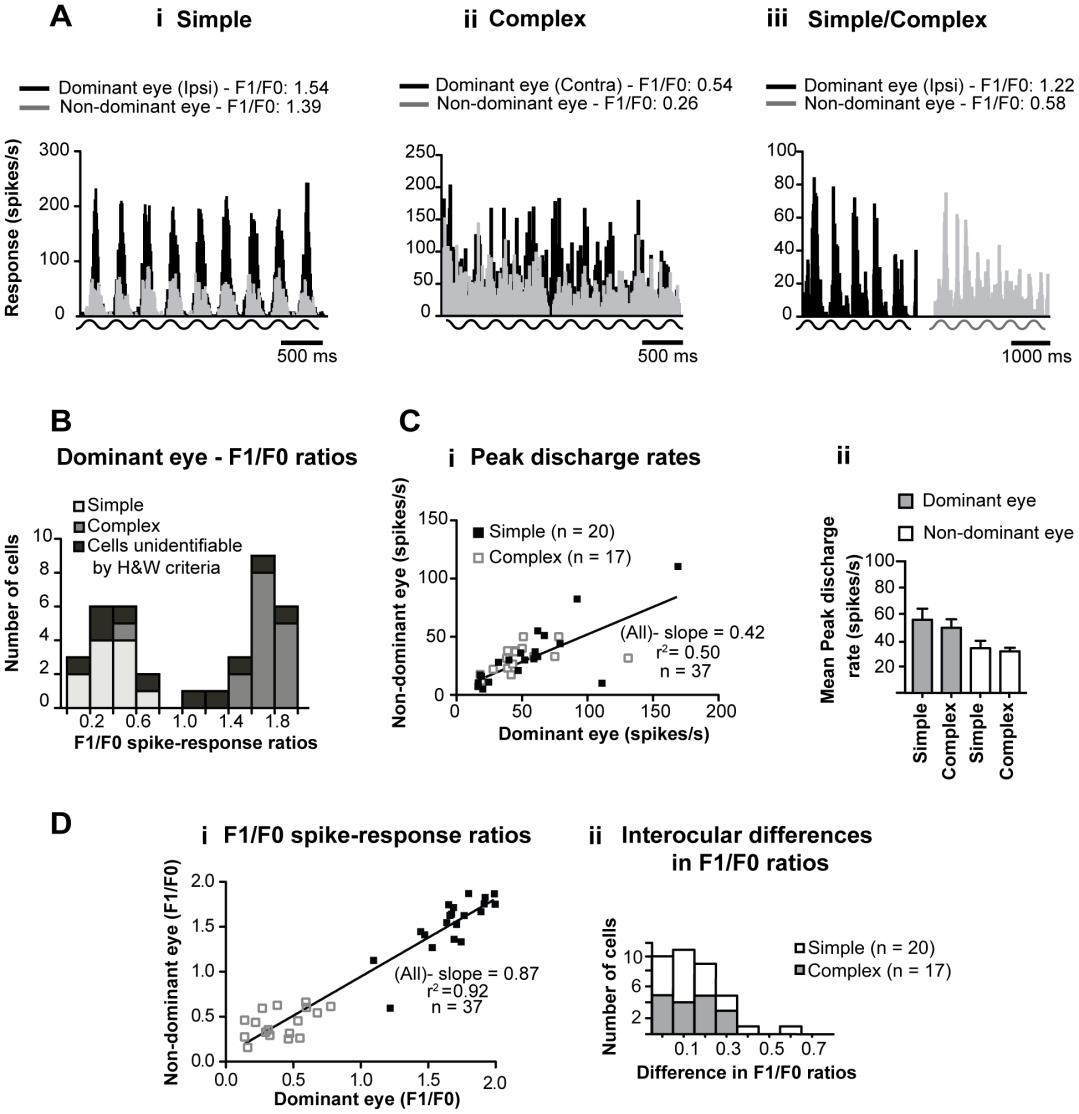
Differentiating between simple and complex cells. **A** Examples of peristimulus time histograms (PSTH) of responses to optimized (orientation, direction of movement, spatial and temporal frequencies, size) achromatic grating patches presented via dominant or non-dominant eyes. Histograms in **Ai** and **Aii** illustrate excellent interocular ‘matching’ of F1/F0 spike-response ratios to high-contrast grating patches optimized for each eye. Histograms in **Aiii** illustrates poor interocular matching of F1/F0 spike-response ratios. **B** Frequency histogram of F1/F0 spike-response ratios for the current sample of binocular parastriate cells to optimized grating patches presented via the dominant eyes. Note that the majority of cells that were identified as simple or complex on the basis of their F1/F0 spike-response ratios were also identified as simple or complex on the basis of Hubel and Wiesel's (1962) criteria. **Ci** Scatter plot of the magnitudes of peak discharge rates for the current sample of cells to optimized grating patches presented via the dominant vs. those to the optimized grating patches presented via the non-dominant eyes. **Cii** Histogram of the mean peak discharge rates of simple and complex cells to optimized drifting gratings presented via the dominant and the non-dominant eyes. Error bars indicate SEM. **Di** Scatter plot of F1/F0 spike-response ratios for optimized stimuli presented via the dominant eyes vs. those for optimized stimuli presented via the non-dominant eyes. **Dii** The frequency histogram illustrates the range of interocular differences in phase-sensitivity (F1/F0 spike-response ratios) elicited individually by optimized grating patches presented via the dominant and non-dominant eyes.

All single neurons recorded from were identified as either simple (20/37; 54%) or complex (17/37; 46%) on the basis of their phase sensitivities expressed as F1/F0 spike-response ratios (simple - F1/F0 ratios >1; complex - F1/F0 ratios <1) to high-contrast, optimized, sRF-confined grating patches presented via the dominant eye (Figs 1Ai and 1Aii). Note that, in Fig. 1B there are two clear peaks in the frequency histogram of F1/F0 spike-response ratios of the present sample [29]. The larger peak is due to prevalence of simple cells with F1/F0 spike-response ratios in the range of 1.6–2.0 while smaller peak is due to prevalence of complex cells with F1/F0 spike-response ratios in the range 0.2–0.6. Consistent with this, the mean F1/F0 spike-response ratio of simple cells for optimized grating patches presented via the dominant eye was 1.674 (SEM ±0.053; range: 1.095–1.995; n = 20) while that of complex cells was 0.406 (SEM±0.05; range: 0.139–0.777; n = 17).

The great majority of cells (15/20; 75%) identified as simple on the basis of their F1/F0 spike-response ratios >1 were also identified as simple on the basis of one of the original criteria put forward by Hubel and Wiesel (1962) [53]. In particular, when optimally oriented, stationary, flashing light bars and/or optimally oriented moving light and/or dark bars were presented via the dominant eye, the receptive fields of these cells contained spatially distinct (non-overlapping) and mutually antagonistic ON and OFF discharge sub-regions [29], [54]. Similarly, in a majority (11/17; 64.5%) of cells identified as complex on the basis of their F1/F0 spike-response ratios <1, receptive fields contained spatially overlapping ON and OFF discharge regions [29], [53], [54]. However, one cell with a complex-like F1/F0 spike-response ratio of 0.54, had spatially non-overlapping ON and OFF discharge regions in its receptive field and thus according to the Hubel and Wiesel's criteria would have been identified as simple (Fig.1B). Furthermore, we were unable to identify a substantial proportion (10/37; 27%) of cells as either simple or complex using the above-mentioned Hubel and Wiesel's criteria. Thus, although these cells generated spike-responses when optimally oriented bars either darker (8/37; 21.5%) or brighter (2/37; 5.5%) than the background moved through their discharge fields [29], they generated very few, if any, action potentials when stationary, flashing, optimally oriented, high-contrast, light bars were used.

### 3.2 Peak firing rates to optimized stimuli

The slope of the linear regression line of peak firing rates to optimized stimuli presented via the dominant eyes vs. those to the stimuli presented via the non-dominant eyes was low (Fig.1Ci). The mean peak firing rates of simple cells to optimized sRF-confined, high-contrast stimuli presented via the dominant (54.8±8.4 spikes/s; range: 16.35–169.0 spikes/s) or non-dominant (33.0±5.7 spikes/s; range: 5.0–105.0 spikes/s) eyes, were slightly higher than those (dominant eyes - 48.65±6.5 spikes/s; range: 18.0–131.0 spikes/s; non-dominant eyes - 30.75±2.65 spikes/s; range: 12.0–50.0 spikes/s) of complex cells (Fig. 1Cii). Due to the exclusion of cells with peak firing rates to optimized stimuli presented via the dominant eyes of <15 spikes/s, the mean peak firing rates to stimuli presented via the dominant eyes were substantially higher than those of area 18 neurons collected in our previous sample [29].

### 3.3 Interocular correlation of phase-sensitivities

In the great majority (35/37; 86.5%) of cells, the phase-sensitivity to high-contrast, sRF-confined, optimized grating patches presented via the dominant eye hardly differed from the phase-sensitivity to such stimuli presented via the non-dominant eye (interocular differences in F1/F0 spike-response ratios ≤0.3; Figs 1Ai, 1Aii, 1Di and 1Dii). Indeed, only one cell (Fig. 1Aiii) exhibited simple-like phase sensitivity (F1/F0 spike-response ratio - 1.22) to optimized grating patch presented via one (the dominant) eye and complex-like phase sensitivity (F1/F0 spike-response ratio 0.58) to optimized grating patch presented via the other (non-dominant) eye. Not surprisingly, there was a very strong, positive correlation between the F1/F0 spike-response ratios to optimized grating patches presented separately via each eye (Fig. 1D, Table 1.1.- Spearman coefficient r  = 0.8971; P<0.0001; n = 37).

**Table 1:** Interocular comparison of receptive field properties of binocular Area 18 neurons (present study; dominant vs. non-dominant eyes).

Properties	Interocular correlation (Spearman correlation coefficient)	Significance of correlation	Number of cells
1. Phase sensitivity (F1/F0 ratio)	r = 0.8971	P<0.0001	37
2. Optimal orientation	r = 0.8858	P<0.0001	37
3. Direction selectivity index	r = 0.7005	P<0.0001	37
4. Optimal spatial frequency	r = 0.7854	P<0.0001	37
5. Spatial frequency bandwidth	r = 0.4050	P<0.0132	30
6. Optimal temporal frequency	r = 0.4307	P = 0.0039	37
7. Summation area	r = 0.2870	P<0.0850	37
8. Suppression index	r = 0.4585	P<0.0022	37

### 3.4 Interocular correlation of orientation/axis of movement preferences

**Figure 2:**
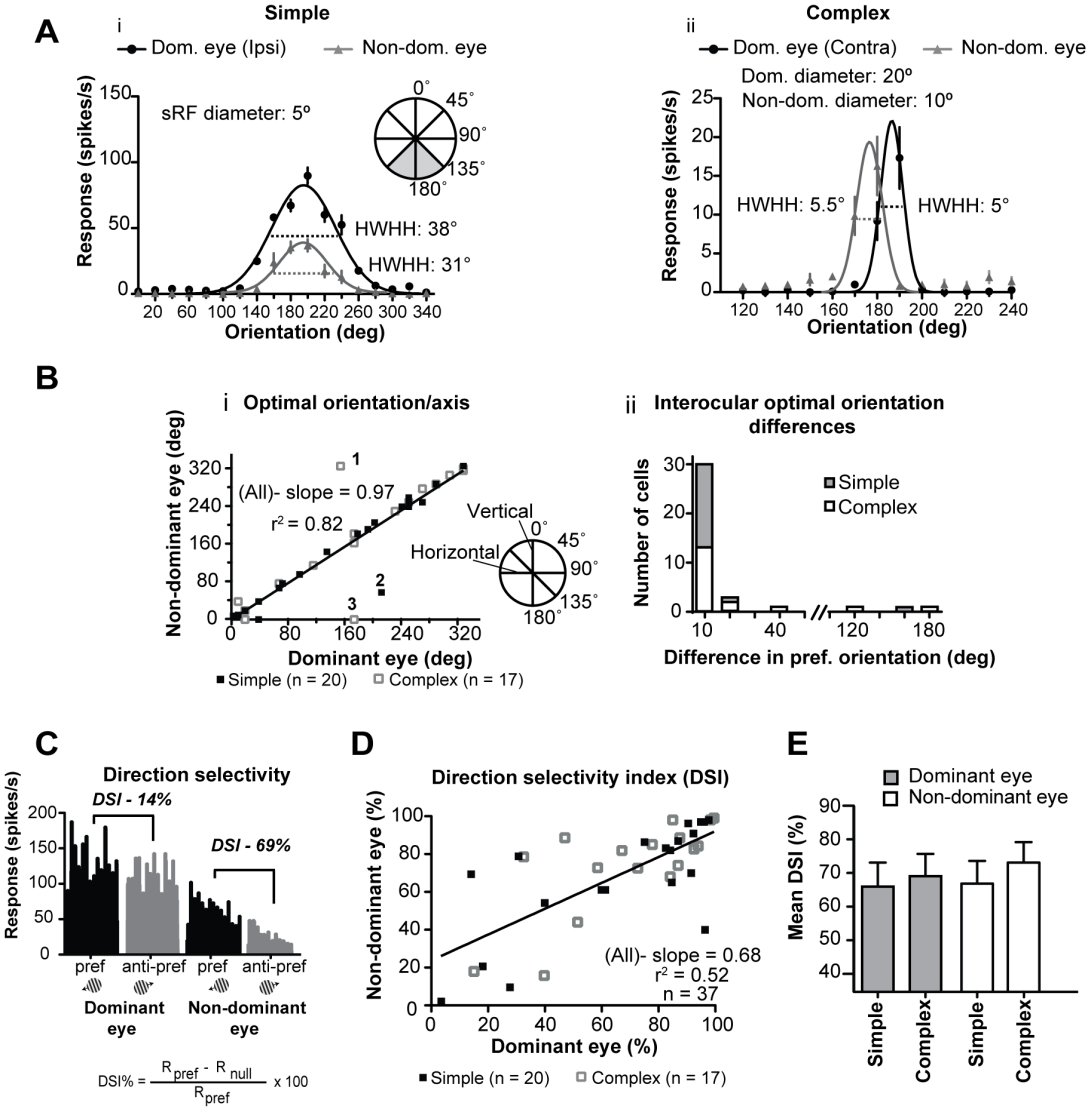
Orientation and direction selectivity. **A** Examples of orientation tuning curves for optimized grating patches presented via dominant and non-dominant eyes. The simple cell in **Ai** has identical preferred orientation for stimuli presented via the dominant and the non-dominant eyes, however the orientation-tuning curve for the dominant eye is broader (greater Half-Width at Half-Height – HWHH) than that for the non-dominant eye. By contrast, in the case of the complex cell in **Aii** while the preferred orientations for stimuli presented via the dominant and non-dominant eyes differ by 20°, the HWHH of the orientation tuning curves are virtually identical. Note that in cells indicated by numbers 1, 2 and 3, the preferred orientation/axis in each eye differ by ∼180°. **Bi** Scatter plot illustrating the interocular matching of optimal orientations; linear regression of the whole sample indicates excellent orientation matching. Inset indicates our convention for defining the orientation of the stimuli; Horizontal - 90° Vertical – 0°. **Bii** Frequency histogram showing interocular differences in optimal orientation for simple and complex cells. **C** Peristimulus time histograms illustrate the responses of a ‘typical’ complex cell to optimized grating patches moving in the preferred and anti-preferred direction for stimuli presented via the dominant and non-dominant eyes. **D** Scatter plot illustrating interocular matching of direction selectivity indices (DSI). **E** Histogram of the mean DSI of simple and complex cells to optimized drifting grating patches presented via the dominant and non-dominant eyes. Error bars indicate SEM.

The majorities of both simple (17/20; 85%; Figs 2Ai and 2B) and complex (13/17; 76.5%; Fig. 2B) cells exhibited excellent interocular optimal orientation/axis of movement ‘matching’- with interocular differences in preferred orientations not exceeding 10° (Fig. 2B). Indeed, only in a small proportion (simple - 3/20; 15%; complex - 4/17; 23.5%) of cells, the interocular differences in the optimal orientation/axis of movement were ≥20° (Figs 2Aii and 2B). Thus, overall interocular correlation of optimal orientations/axis of movement was very strong (Fig. 2B, Table 1.2. - Spearman coefficient r = 0.8858; P<0.0001; n = 37).

### 3.5 Direction selectivity indices

Consistent with previous studies [29], when the optimally oriented optimized grating patches were presented via the dominant eyes, the substantial majority (25/37, 68%) of cells exhibited strong direction selectivity (DSI ≥60%) with very few (4/37, 11%; 3 simple, 1 complex) exhibiting very weak (DSI ≤25%) direction selectivity (Fig. 2C). The slope of the linear regression line of DSI for stimuli presented via dominant eyes vs. DSI for stimuli presented via the non-dominant eyes was relatively low (Fig. 2D). When optimized grating patches were presented via the dominant eyes, the mean DSI of simple (66.35±15%; range: 3.4–97.7%; n = 20) and complex (70.0±6.1%; range: 15.0–99.5%; n = 17) cells were very similar to those (simple: 67.5±6.55%; range: 2.1–98.0%; complex: 73.5±6.05%; range: 15.8–99.0%) for stimuli presented via the non-dominant eyes (Fig. 2E). Although, a substantial proportion of cells exhibited high DSI when optimized stimuli were presented via one eye and low DSI when such stimuli were presented via the other eye (Fig. 2D), the interocular correlation of DSI was strong (Table 1.3. -Spearman coefficient: r = 0.7005; P<0.0001; n = 37). However, consistent with previous reports [36], [37], in some cells in which the preferred orientations were horizontal, the preferred direction for stimuli presented via the dominant eye was opposite (180^o^) or almost opposite to that for stimuli presented via the non-dominant eye (Figs 2Bi and 2Bii).

### 3.6 Optimal spatial frequencies

**Figure 3:**
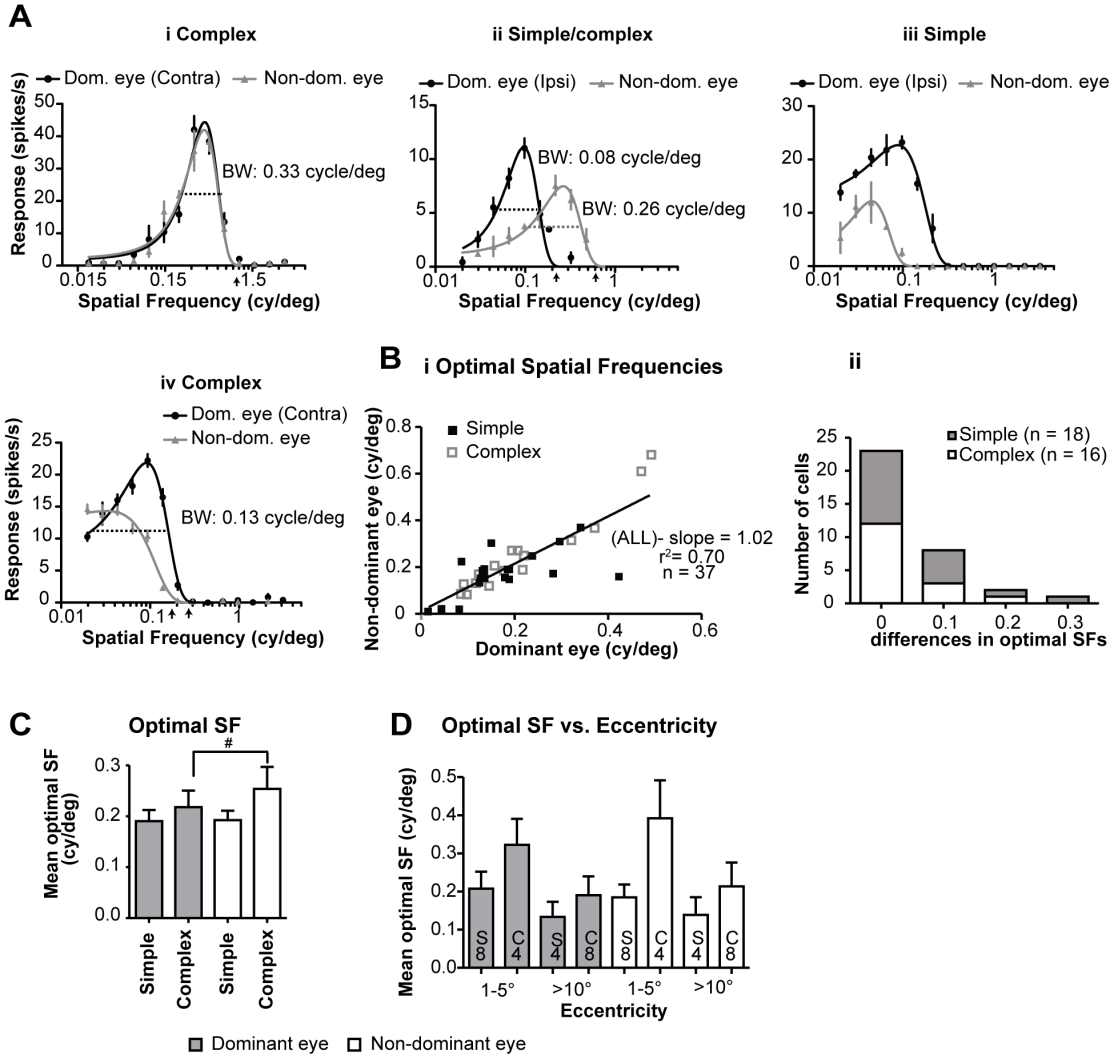
Spatial frequency tuning. **A** Examples of spatial frequency tuning plots of area 18 cells for stimuli presented via the dominant and non-dominant eyes. The bandwidth represents the spatial frequency range which elicits >50% of maximal firing rate. Scatter plot in **Bi** illustrates the interocular matching of optimal spatial frequencies. Black line indicates linear regression line. **Bii** Frequency histogram of interocular differences in optimal spatial frequencies. **C** Histogram illustrating the mean optimal spatial frequencies of simple and complex cells to optimized drifting gratings presented via the dominant and non-dominant eyes. # Indicates a marginal significant (P<0.05, Mann-Whitney test, one-tailed criterion) difference. **D** Histogram showing the mean optimal spatial frequencies for stimuli presented via the dominant or non-dominant eyes for groups of simple (S) and complex (C) based on eccentricity of location of their discharge fields.

Consistent with previous reports [29], irrespective of the eye through which the stimuli were presented, the great majority of area 18 cells (30/37; 81%), were spatial frequency-tuned (Figs 3Ai and 3Aii). A proportion of cells however, exhibited low-pass spatial frequency tuning [29] either irrespective of the eye through which the stimuli were presented (Fig. 3Aiii - 2 simple, 1 complex;) or only when the stimuli were presented via a particular (dominant or non-dominant) eye (Fig. 3Aiv-3 complex, 1 simple). Nevertheless, in the majority of low-pass tuned neurons (Fig. 3Aiii) it was possible to determine the optimal spatial frequency for stimuli presented via either eye (cf. however Fig. 3iv).

In the clear majority (23/34; 65.5%; Figs 3Ai, 3Bi, 3Bii) of cells, the interocular differences in preferred spatial frequencies were very small (Figs 3Bi, 3Bii). The slope of the linear regression line of optimal spatial frequencies for dominant eyes vs. optimal spatial frequencies for the non-dominant eyes was close to 1 (Fig. 3Bi) and the interocular correlation of optimal spatial frequencies was strong (Table 1.4.-Spearman coefficient r = 0.7854; P<0.0001; n = 37).

In case of simple cells the mean optimal spatial frequency for optimized sRF-confined stimuli presented via the dominant (0.174±0.022 cy/deg; range: 0.015–0.423 cy/deg; n = 20) eyes was virtually identical to that (0.175±0.020 cy/deg, range: 0.010–0.370 cy/deg) for the stimuli presented via the non-dominant eyes (Fig. 3C). Consistent with our previous study [29], the optimal spatial frequencies of complex cells tend to be higher than those of simple cells (Fig. 3C). Furthermore, the mean optimal spatial frequency for optimized stimuli presented via the dominant eyes at 0.210 cy/deg (±0.031 cy/deg; range: 0.084–0.492 cy/deg; n = 17) was marginally significantly (P<0.05, Wilcoxon test, one-tailed criterion) lower than that (0.240±0.043 cy/deg; range: 0.020–0.68 cy/deg) for optimized stimuli presented via the non-dominant eyes.

Consistent with numerous previous studies, when the stimuli were presented via the dominant eyes, the optimal spatial frequencies of simple (mean 0.207±0.044 cy/deg; range: 0.081–0.423 cy/deg) or complex (mean: 0.322±0.067 cy/deg; range: 0.206–0.492 cy/deg) cells with centre of the discharge fields located centrally (within 5° from the *areae centrales*) tended to be higher than those (simple - mean: 0.133±0.039 cy/deg, range: 0.081–0.237 cy/deg; complex - mean: 0.179±0.044 cy/deg; range: 0.084–0.471 cy/deg) of neurons with more peripherally (10–20° from the *areae centrales)* located discharge fields (Fig. 3D). Similarly, when the stimuli were presented via the non-dominant eyes - the mean optimal spatial frequency of complex (0.399±0.099 cy/deg; range: 0.250–0.680 cy/deg) and simple (0.184±0.034 cy/deg; range: 0.200–0.370 cy/deg) cells with discharge fields located centrally were substantially higher than those of complex (0.192±0.059 cy/deg; range: 0.020–0.610 cy/deg) or simple (0.138±0.046 cy/deg; range: 0.022–0.247 cy/deg) neurons with discharge fields located more peripherally. Thus, the significant differences in the mean optimal spatial frequencies between the simple and complex cells are not due to overrepresentation in our sample of complex cells with centrally located discharge fields.

### 3.7 Spatial frequency bandwidth

Apart from the minority (7/37–19%) of neurons that exhibited low-pass spatial frequency tuning when the stimuli were presented via one or both eyes, it was possible to assess the spatial frequency-tuning bandwidth (SF-bandwidth) for stimuli presented via either eye (Figs 3Ai and 3Aii).

**Figure 4:**
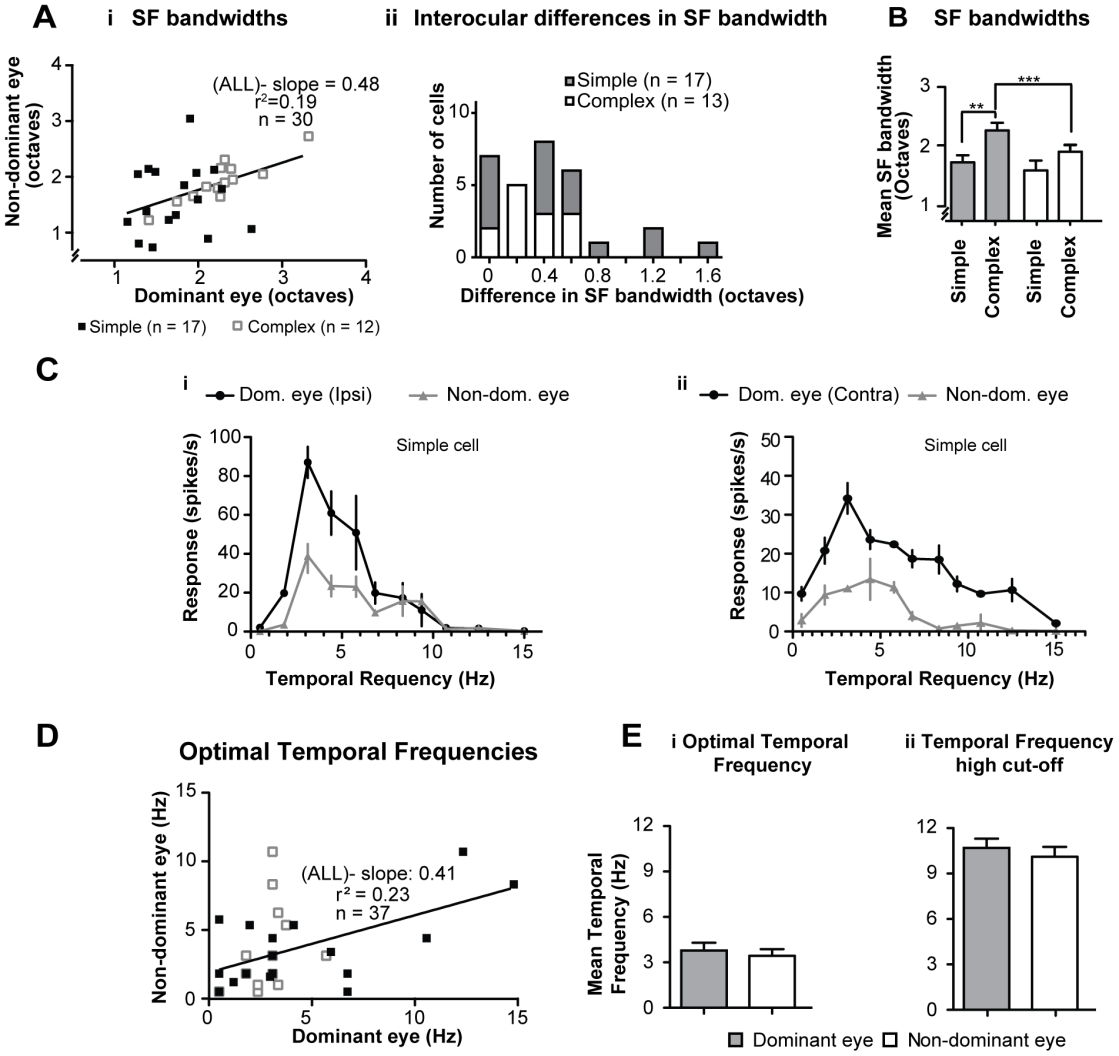
Spatial frequency cut-offs and temporal frequency tuning. **Ai** Scatter plot illustrating the interocular matching of spatial frequency bandwidths; black line indicates linear regression line. **Aii** Frequency histogram of the interocular matching of SF bandwidths for simple and complex cells. **B** Histogram of the mean spatial frequency bandwidths of simple and complex cells for stimuli presented via dominant and non-dominant eyes. ** Indicates significant (P<0.01 Mann-Whitney test) difference; *** indicates significant (P<0.0005, Wilcoxon test) difference. **C** Examples of temporal frequency tuning curves for optimized (orientation, spatial frequency, size) gratings presented via dominant and non-dominant eyes. Note that in case of cell whose responses are illustrated in **Ci** there is an excellent interocular match of both optimal and high cut-off temporal frequencies. However, in case of cell whose responses are illustrated in **Cii** there are clear interocular differences in both optimal and high cut-off temporal frequencies. **D** Scatter plot showing the interocular matching of optimal temporal frequencies for the present sample of area 18 cells. **E** Histograms in **i** and **ii** illustrate respectively the optimal and high cut-off temporal frequencies for stimuli presented via the dominant and non-dominant eyes. Error bars indicate SEM.

The slope of the linear regression line of SF- bandwidths for stimuli presented via dominant vs. SF- bandwidths for stimuli presented via the non-dominant eyes was rather low (Fig. 4Ai). Furthermore, in most cells, there were substantial interocular differences in the SF-bandwidth (Fig.4Aii) and overall the interocular correlation of SF-bandwidth was relatively weak but significant (Table 1.5. - Spearman coefficient r = 0.4050, P<0.0132; n = 30).

The mean SF-bandwidth for optimized, sRF-confined, stimuli presented via the dominant eyes of simple cells at 1.76 octaves (±0.10; range: 1.15–2.64 octaves; n = 17) was significantly (P<0.05, Mann-Whitney test) narrower than that (2.27±0.12 octaves, range: 1.41–3.32 octaves; n = 13) for complex cells (Fig. 4B). In the case of the non-dominant eyes, the SF-bandwidths for both simple (mean - 1.62±0.14 octaves) and complex (mean - 1.92±0.10 octaves) cells were narrower than those for their dominant eye counterparts. However, only in complex cells, the difference between the dominant and non-dominant eyes was significant (Fig. 4B - P<0.0005, Wilcoxon test).

### 3.8 Optimal temporal frequencies and temporal frequencies high cut-offs

In most (but not all- Fig 4Ci) cells there were clear interocular differences in optimal temporal frequencies (Figs 4Cii and 4D) and in small proportions of those cells (simple: 4/20; 20%; complex: 2/17; 12%) the interocular differences exceeded 5 Hz (Fig. 4D). The slope of linear regression line of optimal temporal frequencies for the dominant eyes vs. optimal temporal frequencies for the non-dominant eyes was rather shallow (Fig. 4D). The interocular correlation of optimal temporal frequencies was relatively weak but significant (Table 1.6.- Spearman coefficient r = 0.4307; P = 0.0039; n = 37).

When optimized stimuli were presented via the dominant eyes, the mean optimal temporal frequency of 3.78 Hz (±0.51; range; 0.5–15 Hz) was slightly but not significantly (P>0.1; Mann-Whitney test, one-tailed criterion) greater than that (3.44±0.44 Hz; range: 0.5–10.71 Hz) for stimuli presented via the non-dominant eye (Fig. 4Ei).

When the stimuli were presented via the dominant eyes, the mean temporal frequency high cut-off (Fig. 4Eii) of 10.69 Hz (±0.61; range: 3–15 Hz) was marginally higher than the mean temporal frequency high cut-off for stimuli presented via the non-dominant eye (10.11±0.65 Hz; range: 1–15 Hz).

### 3.9 Sizes of summation receptive fields (sRF)

**Figure 5:**
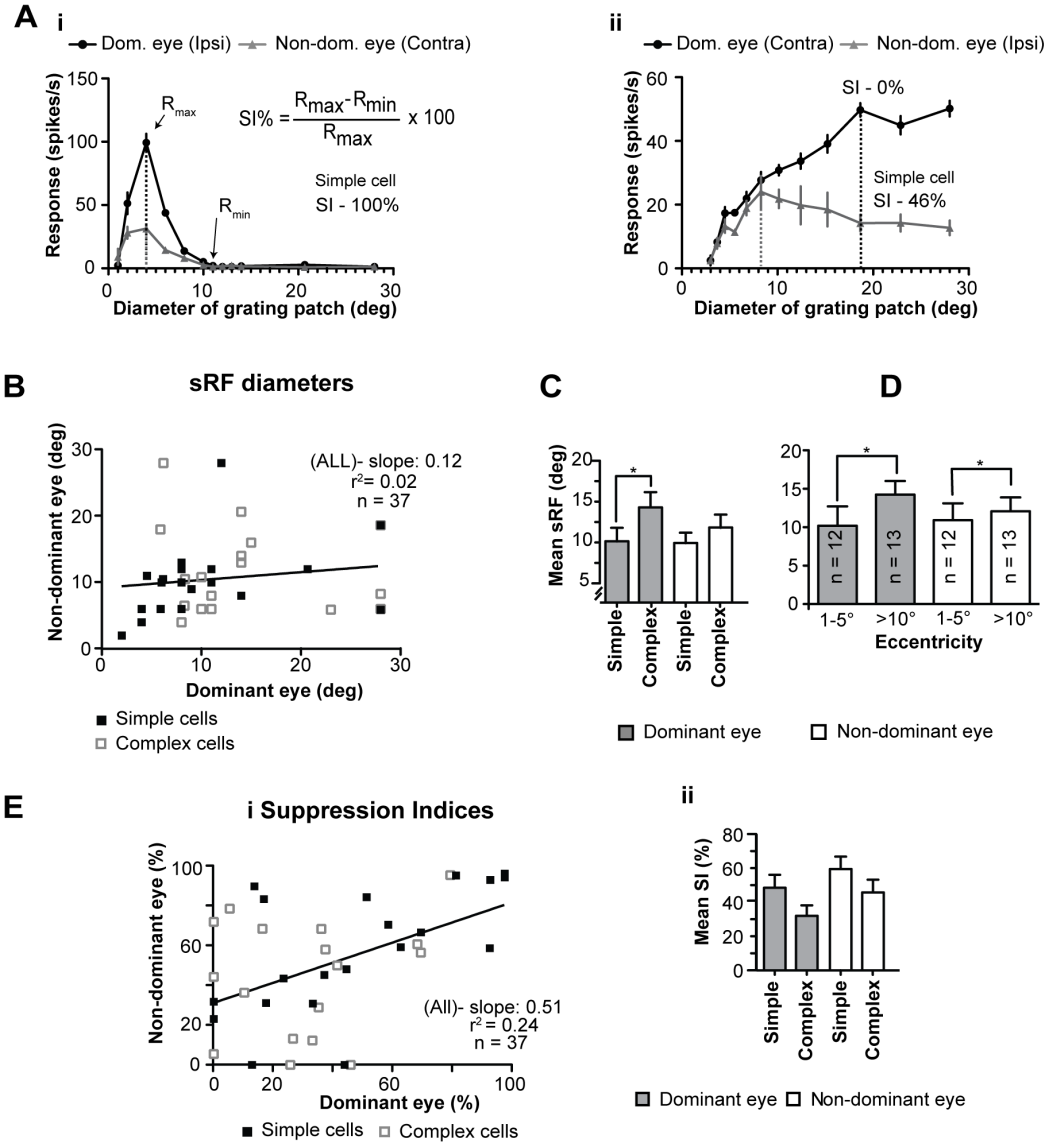
Receptive field sizes and suppression indices. **A** Examples of summative excitatory receptive field (sRF) tuning curves whereby the optimal sRF is the size of the grating patch which elicits maximal spike-response for optimized (orientation, spatial and temporal frequencies) stimuli presented via the dominant and non-dominant eyes respectively. **Ai** illustrates a cell that exhibits good interocular matching of both the sizes of sRF and the strength of surround suppression – high suppression index (SI). Conversely **Aii** exhibits poor interocular matching for both size of sRF and SI. Scatter plot in **B** illustrates the interocular matching of sRF, black line indicate linear regression line. **C** Histogram illustrating the mean sizes of sRF of simple and complex cells for optimized stimuli presented via the dominant and non-dominant eyes; * indicates significant (P<0.05 Mann-Whitney test) difference. **D** Histogram detailing the mean sRF sizes for the dominant and non-dominant eyes with the sample divided on the basis of eccentricity of the location of the discharge fields; * indicate significant (P<0.05, Mann-Whitney test) differences. Scatter plot in **Ei** illustrates the interocular matching of suppression indices (SI) of area 18 cells. Histogram in **Eii** illustrates the mean SI of simple and complex cells for stimuli presented via the dominant and non-dominant eyes respectively.

In some cells the sizes of summation receptive fields (sRF) revealed by stimuli presented via the dominant and those revealed by stimuli presented via the non-dominant eyes were identical (Figs 5Ai and 5B). In many others however, the sRF revealed by stimuli presented via the dominant and non-dominant eye were very different (Figs 5Aii and 5B).

The slope of the linear regression line of diameters of sRF when the stimuli were presented via dominant eyes vs. diameters of sRF when the stimuli were presented via non-dominant eyes was shallow (Fig. 5B) and the interocular correlation of sRF diameters was weak and not significant (Table 1.7. - Spearman coefficient r = 0.2870; P<0.0850; n = 37).

The mean diameter of sRF of simple cells when high-contrast optimized stimuli were presented via the dominant eyes at 10.1° (±1.65°; range: 2.0°–28.0°; n = 20) was virtually identical to that (9.9°±1.3°; range: 2.0°–28.0°) when such stimuli were presented via the non-dominant eyes (Fig.5C). On the other hand, consistent with our previous report [29], the mean diameter of sRF of complex cells revealed by high-contrast, optimized stimuli presented via the dominant eye at 14.3° (±1.85°; range: 5.9°–28.0°; n = 17) was significantly (P<0.05; Mann-Whitney test) larger than that of simple cells (Fig. 5C).

As indicated in Figure 5D and consistent with numerous previous studies, irrespective of the eye (dominant or non-dominant) through which the stimuli were presented, the sRF of cells with centre of the discharge fields located centrally (within 5° from *area centralis*) tended to be smaller (dominant eyes: mean 10.2°±2.5°; range: 2–28°; n = 12; non-dominant eyes: 10.9°±2.2°; range: 2–28°) than those (dominant eyes: mean 13.1°±1.65°; range: 6.2–28°; n = 13; non-dominant eyes: 12.4°±1.75°; range: 6.2–28°) of cells with the discharge fields located more peripherally (>10° from *area centralis*). Furthermore, in both cases (dominant and non-dominant eyes) the differences are significant (P<0.05; Mann-Whitney test).

### 3.10 Suppression indices

In most area 18 cells, irrespective of the eye through which the stimuli were presented, extending the diameter of optimized grating patches beyond the summation receptive field resulted in a substantial reduction (or even a complete abolition - suppression index 100%) in the magnitude of spike-responses (Fig 5Ai).

In some cells however, the reduction in the magnitude of spike-responses with increasing the size of the grating patches was apparent only when the stimuli were presented via one, but not the other eye (e.g. Fig. 5Aii). Furthermore, in a proportion of cells extending the stimulus presented through either eye did not result in a significant reduction in the magnitude of spike response (SI - 0%; Fig. 5Ei).

The slope of linear regression line of SI of dominant vs. non-dominant eyes was not steep (Fig. 5Ei) and the interocular correlation of SI was not strong but significant (Table 1.8. - Spearman coefficient r = 0.4585; P<0.0022; n = 37).

The mean SI for simple cells when the stimuli were presented via the non-dominant eyes (59.5±7.25%; range: 0.0–100%; n = 20) was higher (but not significantly - P>0.1; Mann-Whitney test, one-tailed criterion) than that (48.55±7.55%; range: 0.0–100%) when the stimuli were presented via the dominant eyes (Fig. 5Eii). However, in the case of complex cells, the mean SI (45.8±7.5%; range: 0.0–99.2%; n = 17) when the stimuli were presented via the non-dominant eyes was significantly (P<0.002; Mann-Whitney test) *higher* than that (32.0±6.15%; range: 0.0–81.35%) when the stimuli were presented via the dominant eyes (Fig. 5Eii).

### 3.11 Contrast sensitivities

**Figure 6:**
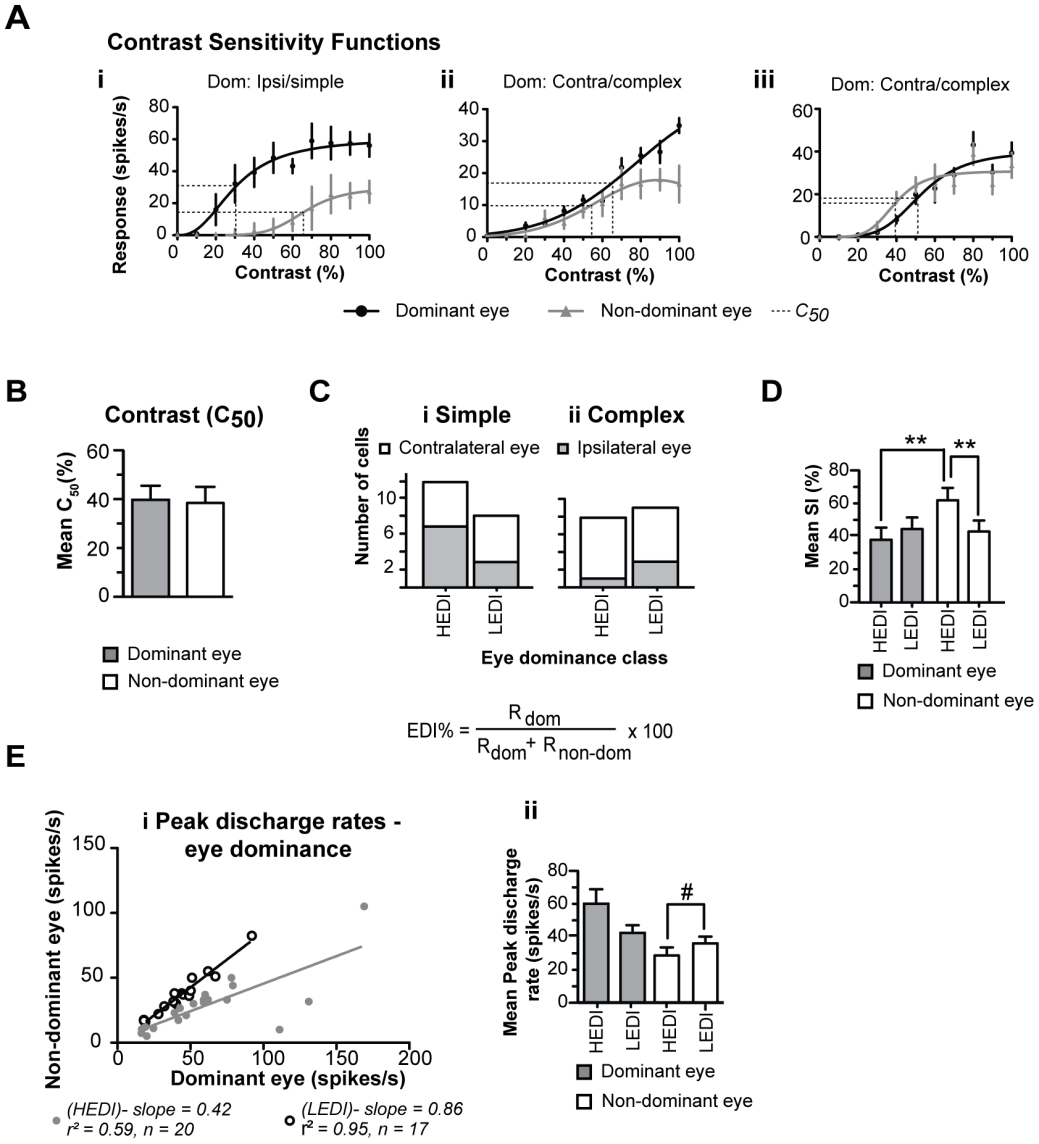
Contrast sensitivity and comparison of high vs. low eye dominance. **A** Examples of contrast response functions for optimized grating patches presented via the dominant and non-dominant eyes. Note that in some cells (**Ai**) irrespective of the contrast, the response to stimuli presented via non-dominant eye is much weaker than that to stimuli presented via dominant eye. In other cells however, at low contrasts the response to stimuli presented via the dominant eye is not much stronger than that to stimuli presented via the non-dominant eye (**Aii**) or the eye dominance is reversed (**Aiii**). The C_50_ contrasts, that is, the contrasts at which the magnitude of response to optimized stimuli confined to the sRF reached 50% of maximum response are indicted by the dashed lines. **B** Histogram showing the mean C_50_ values of the contrast response functions of the current sample of cells when stimulated via the dominant or non-dominant eyes. **C** Frequency histograms of cells with high eye dominance indices (HEDI) and cells with low eye dominance indices (LEDI). Also shown is the equation by which eye dominance index has been determined. **D** Mean SI of HEDI and LEDI cells for stimuli presented via the dominant or non-dominant eyes. Error bars indicate SEM; ** indicate significant (P<0.01, Wilcoxon test) differences. **Ei** Scatter plot of the peak discharge rates for optimized grating patches presented via the dominant vs. optimized grating patches presented via the non-dominant eyes. **Eii** Mean peak discharges rates of HEDI and LEDI cells for stimuli presented via the dominant or non-dominant eyes. Error bars indicate SEM; # indicates marginally significant (P<0.05, Mann-Whitney test, one-tailed criterion) difference.

In over two-thirds of the sample (24/35; 68.5%), the response saturated when high contrast stimuli (≥80%) were presented via either eye (Figs 6Ai & 6Aii). In the remainder (11/35; 31.5%) however, the response did not saturate even at 100% contrast. In all but one cell, the lack of contrast saturation was apparent only when the stimuli were presented via one eye (the non-dominant eye, 4 cells; Fig. 6Aii; the dominant eye, 6 cells; Fig. 6Aiii).

For most cells, the mean C_50_ contrast, that is, the contrast at which the magnitude of response to optimized stimuli confined to the sRF reached 50% of maximum response, was quite similar irrespective of the eye through which the stimuli were presented. Indeed, the mean C_50_ contrast (49.9%; SEM±2.9%; range: 20.2–89.5%; n = 37) for optimized stimuli presented via the dominant eyes was almost identical to that (49.25%; SEM±3.3%; range: 11.05–85.15%; n = 35) for optimized stimuli presented via the non-dominant eyes (Fig. 6B).

### 3.12 Eye dominance and suppression indices

At saturating or 100% contrasts, a large proportion of cells (17/37; 46%) were only weakly (Eye dominance index - EDI≤60%, see Materials and methods; cf. [40] dominated by either the contralateral (11/23; 48%; 5 simple, 6 complex) or the ipsilateral (6/14; 43%; 3 simple, 3 complex) eyes. These cells are defined here as low eye dominance index (LEDI) cells (Fig. 6C; cf. combined classes 3, 4 and 5 cells of [53], [55]. The cells in which the EDI exceeded 60% are defined here as cells with high eye dominance index (HEDI). In a majority (12/20; 60%) of HEDI cells, the contralateral eye was strongly dominant (Fig. 6C; cf. class 2 cells of [53], [55]–[57]). In the remainder (8/20; 40%) of HEDI cells, the ipsilateral eye was strongly dominant (Fig. 6C; cf. class 6 cells of [53], [55]–[57]). It is interesting to note in this context that in a few cases (6/35), the eye dominance was contrast dependant and the eye which appeared to be dominant when the high contrast (≥80%) stimuli were used, became non-dominant when the lower contrast stimuli were used (e.g. Fig. 6Aiii). With one exception, this contrast dependent reversal of eye dominance was apparent only in LEDI cells.

The mean SI when stimuli were presented via the dominant eyes of HEDI cells (37.95±7.4%; range: 0.0–100%; n = 20) was slightly and not significantly (Table 2) lower than that (44.45±7.0%; range: 0.0–100%; n = 17) of LEDI cells (Fig. 6D). Although the mean peak discharge rate of HEDI cells to optimised stimuli presented via the dominant eyes (60.1±8.9 spikes/s: range: 16.35–169 spikes/s) was substantially higher than that (42.4±4.65 spikes/s: range: 18.0–92.0 spikes/s) of LEDI cells (Fig. 6Ei), the difference was not significant (Table 2).

**Table 2:** Magnitude of suppression indices of binocular area 18 neurons (HEDI vs. LEDI).

	Mean SI of HEDI cells	Mean SI of LEDI cells	Significance of the differences*
**Dominant eye**	37.95±7.4%; n = 20	44.45±7.0%; n = 17	P>0.05
**Non-dominant eye**	61.9±7.5%; n = 20	43.0±6.7%; n = 17	P<0.02
**Significance of the differences∧**	P<0.02	P>0.05	

*P values refer to result of Mann-Whitney U-test (two-tailed criterion).

∧P values refer to result of Wilcoxon matched-pairs signed-ranks test (two-tailed criterion).

On the other hand, the mean SI revealed by stimuli presented via the non-dominant eyes of HEDI cells was significantly (P<0.02; Mann-Whitney test) higher (61.9±7.5%; range: 0.0–100%; n = 20) than that (43.0±6.7%; range: 0.0–96.7%; n = 17) of LEDI cells (Fig. 6D). Consistent with this, the mean peak discharge rate of HEDI cells to optimised stimuli presented via the non-dominant eyes (28.55±4.9 spikes/s: range: 5.0–105 spikes/s) was substantially *lower* than that (36.0±4.1 spikes/s: range: 16.0–82.5 spikes/s) of LEDI cells (Fig. 6Eii).

## Discussion

There are two important caveats to our conclusions: 1) our sample of area 18 cells was restricted to cells responding strongly (≥15 spikes/s) to optimized stimuli presented via the dominant eye and 2) very few cells in our sample were recorded from the supra or infragranular layers.

### 4.1 Interocular matching of phase-sensitivities

In the previous as well as the current studies in which there was quantative interocular comparison of F1/F0 spike-response ratios of binocular cells in cat's primary visual cortices [40], [41], the interocular differences in the F1/F0 spike-response ratios tended to be small (<0.3). Good interocular match of phase-sensitivities is consistent with very good interocular matching in simple-like vs. complex-like identification based on Hubel and Wiesel's criteria (spatially separate vs. spatially overlapping ON and OFF discharge fields) in areas V1 of the cat [13]–[16], [53], [58] or mouse [59].

In the present sample, the proportion of binocular area 18 cells, in which there was interocular mismatch between simple-like and complex-like F1/F0 spike-response ratios (1/37; 2.5%) was substantially smaller than those in the earlier reports on cat's area 18 (13/69–19%; [40], [41]) or area 17 (10/125–8.0%; [40], [41]). Unlike in the previous studies, very few binocular neurons included in our sample had F1/F0 spike-response ratios close to 1, that is, the value that is defined as the border between complex and simple cells [29], [48], [60].

It is unlikely that strong interocular matching of phase-sensitivities is determined by congruent binocular visual experience. Thus, it has been reported that in visually inexperienced young kittens, those area 17 cells which were orientation selective for stimuli presented via each eye, exhibited the same (according to Hubel and Wiesel's criteria), either simple-like or complex-like characteristics, irrespective of the eye through which the stimuli were presented [61], [62]. Similarly, in binocular V1 cells recorded from small binocular zone of the striate cortex of adult mice, dark-rearing does not affect a good spatial correspondence of separate ON and OFF discharge regions plotted through either eye [59].

### 4.2 Interocular matching of orientation/axis of movement selectivity

It has been reported that in cats under a virtually identical anaesthetic and paralysis regime to that employed in the present study, there is small degree of in-cyclotorsion of the eyes [15]. Despite this, present data indicate an excellent interocular matching of optimal orientations in binocular area 18 neurons. It is very unlikely that the large interocular differences in preferred orientation are due to the large cyclotorsion of the eyes since recording of cells with large (≥20°) interocular differences in preferred orientation was always preceded and followed by recordings of cells with no or small (<10°) interocular differences in preferred orientations. It is worth noting that in area 17 of normal cats, the interocular mismatches in optimal orientation very rarely exceed 15° [15], [40], [63], [64].

Blakemore and his colleagues (1972) suggested that since the interocular orientation differences occur when viewing surfaces slanted in depth, the interocular differences in the preferred orientations of binocular neurons might constitute the basis of a ‘second neural mechanism for depth perception’ [15]. However, as far as the striate cortices of cats [15] and macaque monkeys [64] are concerned, binocular neurons showing interocular orientation disparities are selective for interocular position disparities. In view of the fact that in the present study, the proportion of area 18 cells with high interocular mismatches (>10°; 7/37; 19.0%) of preferred orientations is substantially greater than that (7/74; 9.5%) in area 17 [15], one could argue that this postulated second mechanism of depth perception might ‘kick in’ in area 18 [41], [65], [66].

Several lines of evidence indicate that there is a negative relationship between the large interocular mismatches of preferred orientations and binocular interactions. Thus, unlike in the normally raised cats, in cats that were raised in darkness and for 2–5 hours a day each eye viewed elongated contours of very different orientations (horizontal vs. vertical), only a minority of area 17 cells were selective for orientation. Virtually all cells which were orientation selective were monocular and cells which preferred orientations approximately vertical produced spike-responses only when the stimuli were presented via the eye which viewed vertical contours while cells which preferred orientation approximately horizontal produced spike-responses only when the stimuli were presented via the eye which viewed horizontal contours [67], [68].

In case of binocular neurons in cat's area 17, the degree of interocular matching of optimal orientation is strongly dependent on congruency of binocular experience of visual contours during the so-called critical period of development [62], [69]–[75]. Similarly, while in very young normal mice (postnatal days 20 - 23) 50% of binocular cells recorded from the small binocular zone of the primary visual cortex exhibit very large (>27.5°) interocular mismatches in preferred orientations, in older, visually experienced mice, the interocular mismatches in preferred orientations are much smaller (median 10.4°) [76]. Furthermore, while in ∼80% of binocular V1 cells of visually experienced mice the optimal orientation for stimuli presented via each eye did not differ by >30^o^, in ∼50% of binocular V1 cells of dark-raised mice the optimal orientations for stimuli presented via each eye were mismatched by >30° [59]. It is worth noting that, binocular cells in the primary visual cortices of the mice with their relatively good interocular matching of optimal orientations contribute strongly to binocular integration of retinal disparities and thus are able to extract information relevant to estimation of depth [77].

It appears that in mammalian primary visual cortices, good interocular matches of preferred orientations are essential for facilitatory or just summative binocular interaction at the single neuron level. Indeed, in case of area 17 neurons of cats [24], [63], [78]–[85] or macaque monkeys [86], orientations outside the excitatory tuning range of the cell produce a clear suppression. In cat's primary visual cortices common binocular experience of visual contours is not necessary for development of identical orientation maps for stimuli presented separately through each eye [90]–[92]. Nevertheless, it is very likely that some kind of correlation based ‘Hebbian’ learning mechanism [75], [93], [94] including stimulus-timing-dependent-plasticity [95], [96] is responsible for excellent interocular matching of preferred orientations of binocular cells in mammalian primary visual cortices. Putative mechanisms that underpin the orientation selectivity in mammalian visual cortex are still hotly debated [53], [87]–[89]. The strong interocular correlation of optimal orientations of binocular area 17 and area 18 neurons, suggest that the optimal orientations are, at least partially, determined by the mechanisms operating at post binocular convergence, that is, cortical level.

### 4.3 Direction selectivity indices

It has reported that some area 18 binocular neurons, unlike neurons in area 17, are tuned to direction of motion in depth [36], [37], [97], [98]. Consistent with those findings in a proportion of cells in the present sample, the preferred direction of movement for stimuli presented via the dominant eye was opposite to the preferred direction of drift for stimuli presented via the non-dominant eye. As previously mentioned, such cells respond strongly to stimuli approaching the animal. However, the proportion of such cells was small and the present data does not provide strong support for the notion that area 18 cells are tuned to direction of motion in depth.

Overall, the strong interocular correlation of DS indices of binocular area 18 neurons suggest that the direction selectivities are determined, at least partially, by the mechanisms operating at post binocular convergence, that is, cortical level.

Direction selectivity, like orientation selectivity, is strongly susceptible to the influence of visual experience. Thus, in kittens exposed exclusively to stationary, flashing (8 Hz) contours and therefore deprived of experience of visual motion, there is a dramatic reduction in the number of area 17 cells exhibiting narrow directional tuning [75], [99]. Furthermore, in the kittens raised in uni-directionally moving visual contours, most area 17 cells respond more strongly to stimuli moving in the direction to which the animals were exposed [75], [99]. To our knowledge, there is no data collected from cats raised in visual environment in which each eye was exposed to opposite directions of movement.

### 4.4 Optimal spatial frequencies

Consistent with a somewhat inverse relation between the optimal spatial frequency and eccentricity of receptive field position of geniculate neurons [100], irrespective of the eye through which they were stimulated (dominant or non-dominant), the optimal spatial frequencies of both complex and simple cells with discharge fields close to *areae centrales* tended to be higher than those of cells with more peripherally located discharge fields. It is worth noting that when the stimuli were presented via the non-dominant eyes, optimal spatial frequencies (>0.6 cy/deg) of some complex cells were in the range of X-type rather than Y-type geniculate neurons [100], [101]. In view of paucity of direct X-type geniculate projection to area 18 [28], [47], [51], [52], [102] such putative X-type input would have to be relayed via area 17 [103]–[105], which receives its principal dorsal thalamic input from X-type geniculate neurons and sends massive projections to area 18 [27], [28], [106], [107]. Indeed, consistent with dominance of X-type input to area 17, The present study along with others show that the mean optimal spatial frequencies of both simple and complex cells recorded from area 17 of the cat are ∼2.5-3 times higher than those of area 18 cells [40], [48], [29]. It is also worth noting that while the optimal preferred spatial frequencies of area 17 cells are largely independent of temporal parameters of drifting, in the case of area 18 cells, the optimal spatial frequencies became higher when the temporal frequency of drift or velocity of motion was reduced suggesting that at lower temporal frequencies area 18 cells are driven by X rather than Y-input [108], [109].

### 4.5 Interocular correlations of optimal spatial frequencies

It has been suggested that interocular differences in spatial frequency (spatial frequency disparities), like the interocular differences in the preferred orientations (see above) might also provide information concerning three-dimensional (3D) slant or tilt (away fron the fronto-parallel plane) of surfaces viewed [110]–[112].

It has been reported that interocular ‘mismatches’ in preferred spatial frequencies of binocular area 17 neurons are very common in cats binocularly deprived of visual contours throughout the critical period [73]. In area 17 of normal cats [40] and macaques [113], cells with interocular mismatches in optimal spatial frequencies are reported to be rather uncommon. Indeed, in binocular area 17 cells of normal cats, the interocular correlation of optimal spatial frequencies is very strong (coefficient of correlation - r = 0 92; 40). Others however, report substantial proportions of binocular cells with interocular mismatches in optimal spatial frequencies both in area 17 [41], [114] and area 18 [41] of normal cats. Overall, the strong interocular correlations of optimal spatial frequencies of binocular area 17 and area 18 neurons, suggest that the optimal spatial frequencies are, at least partially, determined by the mechanisms operating at post binocular convergence, that is, cortical level.

In the majority but not all, cat's and macaque's area 17 cells, spatial frequencies on one or both sides of the excitatory spatial frequency tuning range of the cells produce a clear suppression [115]–[117]. On the other hand, silent suppressive surrounds of area 18 cells tend to be more broadly tuned for spatial frequency than the excitatory receptive fields (sRF in the present terminology) but the optimal spatial frequencies of suppressive surround and sRF tend to be the same [29].

### 4.6 Spatial frequency bandwidths

Sharper spatial frequency tuning (narrower bandwidth) of cortical cells in relation to spatial frequency tuning of their geniculate inputs has been attributed to inhibitory intracortical network [116]. Similarly, narrower spatial frequency bandwidths of simple (mean 1.76 octaves for stimuli presented via the dominant eyes) vs. complex (mean 2.27 octaves for stimuli presented via the dominant eyes) area 18 cells might be related to the fact that suppressive indices of simple cells tend to be greater than those of complex cells. On the other hand, in case of binocular area 17 cells, the mean spatial frequency bandwidth of simple cells to stimuli presented via the dominant eye at 1.73 octaves was not significantly different from that (1.80 octaves) of complex cells (40). Furthermore, the reported (40) interocular correlation of spatial frequency bandwitdh of area 17 neurons of normal cats (correlation coefficient r = 0.76) is substantially stronger than that (correlation coefficient r = 0.405) of area 18 cells (the present study).

It is also worth noting that in the case of cat's LGN neurons, iontophoretic application of selective γ- amino-butyric acid, GABA_A_ receptor blocker, bicuculline in most cases results in converting spatial frequency tuned cells into low-pass cells [118].

### 4.7 Temporal frequencies

Area 18 neurons are characterized by lower temporal frequency high cut-offs than those of LGN neurons [29], [100], [101]. The wide range of optimal temporal frequencies of area 18 cells irrespective of the eye (dominant or non-dominant) and poor interocular correlation appears to reflect a great heterogeneity of temporal responses of LGN neurons [100], [101]. To our knowledge, there are no published data concerning the interocular correlation of optimal temporal frequencies and temporal frequencies high-cut offs in binocular area 17 cells of normal cats.

There does not seem to be a good teleological ‘stereoscopic’ reason for imposing good interocular correlation of optimal temporal frequencies. On the other hand, the latency differences between the responses to stimuli presented via dominant eye vs. the non-dominant eye might play an important role in unitary stereoscopic perception [119].

### 4.8 Sizes of sRF

The interocular correlation of sRF sizes was very poor. In our previous study [29] we have provided substantial evidence indicating that the sizes of sRF, as well as the sizes of the discharge fields of area 18 cells are inversely related to the magnitude of the suppressive indices. Thus, the smaller sRF revealed by stimulation via the non-dominant eyes are consistent with greater SI revealed by stimulation via the non-dominant eyes. As mentioned earlier, irrespective of the eyes (dominant or non-dominant) through which the stimuli were presented, the optimal spatial frequencies of both simple and complex cells with centrally located discharge fields tended to be higher than those of cells with more peripherally located discharge fields. Consistent with the inverse relation between dominant eye discharge field sizes and optimal spatial frequencies, sRF of both simple and complex cells with centrally located discharge fields tended to be smaller than those of cells with more peripherally located discharge fields. However, this inverse relationship between the sRF size and the optimal spatial frequency is not consistent when comparing simple and complex cells.

### 4.9 Suppression indices and eye dominance

The magnitude of spike-responses of area 18 neurons is inversely related to the magnitude of SI [29]. Consistent with this, in about 50% of area 17 cells, iontophoretic application of GABA_A_ receptor blocker - bicuculline results in either: 1) conversion of monocular neurons into binocular neurons activated approximately equally strongly by stimuli presented via each eye (our LEDI cells) or 2) reversal of eye dominance of binocular neurons [120]. Furthermore, shift in the ocular dominance of neurons in primary visual cortex of the mouse following the monocular deprivation during the critical period, is based on parallel reduction of both excitation and inhibition driven by the deprived eye as well as reduction of the inhibition (but not excitation) driven by the non-deprived eye [121]. Thus, it appears that in a large proportion of cells in primary visual cortices, the eye dominance is based on the inhibitory process suppressing the non-dominant eye input rather than substantial interocular differences in the strength of the excitatory inputs [122].

### 4.10 Suppression indices and eye dominance columns: putative relation to stereoscopic depth perception

The precise functional micro-architecture for binocular disparity selectivity in cat area 18 appears to be unrelated to the micro-architecture of ocular dominance [123]. However, Gardner & Raiten [124], reported that among neurons recorded from cat area 18 and along the 17/18 border, those with a low eye dominance indices (presumed equivalents of our LEDI cells), tend to be insensitive to binocular position disparity while those with high eye dominance indices (presumed equivalents of our HEDI cells) as well as apparently monocular cells, tend to be highly sensitive to binocular position disparity. We have established here, that at least in the distinct part of cat primary visual cortex, the parastriate cortex, the suppression indices of silent, extra-classical receptive fields of the non-dominant eyes of HEDI cells are significantly greater than those of the dominant eyes. This combined with well established anatomical segregation of eye dominance columns in primary visual cortices (see **Introduction**) would provide neuronal networks with additional information which might play a role in binocular depth discrimination - stereoscopic vision.

## Summary and Conclusions

We have tested quantitatively the degree of interocular matching of receptive field properties in a sample of binocular neurons recorded from cytoarchitectonic area 18 (parastriate cortex) of the cat.

Observed by us good interocular ‘matching’ (strong interocular correlations) of phase-sensitivities and preferred orientations in area 18 (Table 1.1. and 1.2.) is consistent with earlier reports of good interocular matching of phase-sensitivities and preferred orientations in binocular cells recorded from cytoarchitectonic area 17 (striate cortex- see 4.1 and 4. 2 of the Discussion) of normal cats.

There was also a strong interocular correlation of direction selectivity indices (DSI) of area 18 neurons (Table 1.3.). However, consistent with earlier reports in some cells, the preferred direction for stimuli presented via the dominant eye was opposite to this for stimuli presented via the non-dominant eye (sensitivity to looming objects).

There was a strong interocular correlation of optimal spatial frequencies of area 18 neurons (Table 1.4.). The interocular correlation of optimal spatial frequencies of binocular in area 17 neurons of normal cats, is also very strong (see 4.4 and 4.5 of the Discussion). However, in proportions of both simple and complex area 18 and area 17 cells (see 4.5 of the Discussion), the optimal spatial frequencies for stimuli presented via the dominant eyes were different from those for stimuli presented via the non-dominant eyes.

The interocular correlation of spatial frequency bandwidth of area 18 neurons was rather weak (Table 1.5.). Interestingly, the interocular correlation of spatial frequency bandwidth of area 17 neurons was much stronger (see 4.6 of the Discussion).

The interocular correlations of optimal temporal frequencies, size of summation areas of excitatory receptive fields (sRF) and suppression indices (SI) of area 18 neurons was weak (Table 1.6.,1.7.,1.8.).

In area 18 neurons with high eye dominance indices (HEDI cells), the mean magnitudes of suppressions evoked by stimulation of silent, extra-classical receptive fields via the non-dominant eyes, were significantly greater than those when the stimuli were presented via the dominant eyes (Table 2). It is likely, that similar interocular differences characterize HEDI cells in area 17 (see 4.10 of the Discussion).

We argue that as in the case of binocular neurons in cat's area 17, good interocular matching of certain receptive field properties of binocular area 18 neurons, plays important role in creation of stereoscopic single vision as well as extracting from retinal images information necessary for effective binocular depth discrimination.
